# General mixture item response models with different item response structures: Exposition with an application to Likert scales

**DOI:** 10.3758/s13428-017-0997-0

**Published:** 2018-01-10

**Authors:** Jesper Tijmstra, Maria Bolsinova, Minjeong Jeon

**Affiliations:** 1 Department of Methodology and Statistics, Faculty of Social Sciences, Tilburg University, PO Box 90153, 5000 LE Tilburg, The Netherlands; 2 University of Amsterdam, Amsterdam, Netherlands; 3 University of California, Los Angeles, CA USA

**Keywords:** Item response theory, General mixture item response models, Mixture modeling, IRTree models, Measurement invariance, Likert scales, Response styles

## Abstract

This article proposes a general mixture item response theory (IRT) framework that allows for classes of persons to differ with respect to the type of processes underlying the item responses. Through the use of mixture models, nonnested IRT models with different structures can be estimated for different classes, and class membership can be estimated for each person in the sample. If researchers are able to provide competing measurement models, this mixture IRT framework may help them deal with some violations of measurement invariance. To illustrate this approach, we consider a two-class mixture model, where a person’s responses to Likert-scale items containing a neutral middle category are either modeled using a generalized partial credit model, or through an IRTree model. In the first model, the middle category (“neither agree nor disagree”) is taken to be qualitatively similar to the other categories, and is taken to provide information about the person’s endorsement. In the second model, the middle category is taken to be qualitatively different and to reflect a nonresponse choice, which is modeled using an additional latent variable that captures a person’s willingness to respond. The mixture model is studied using simulation studies and is applied to an empirical example.

**Electronic supplementary material:**

The online version of this article (10.3758/s13428-017-0997-0) contains supplementary material, which is available to authorized users.

## Introduction

There are a large number of different item response theory (IRT) models available in the literature (see e.g., Embretson & Reise, 2013; Hambleton & Swaminathan, 1985; Lord & Novick, 1968), allowing one to model dichotomous as well as polytomous response data and to relate these to one or multiple latent variables (Reckase, 2008). These models propose different ways of linking the probability of observing a particular item score to the considered latent variable(s) through the item response function (IRF). This IRF can be parametric or nonparametric in nature, and can be more or less restricted in its shape, depending on the particular IRT model. However, a common assumption shared by most of these models is that whatever the precise specification of the relationship between the observed responses on an item and the attribute(s) in question is, the same IRF is applicable to all persons (Lord & Novick, 1968). This can be seen as imposing a form of measurement invariance (Mellenbergh, 1989; Meredith, 1993; Millsap, 2011) because it assumes that a single IRT model is appropriate for all persons in the sample, and hence that no between-person differences exist with respect to the IRFs. We will call this assumption *IRT measurement invariance* (MI), in order to emphasize that we are considering the assumption that a single IRT model is appropriate for all persons in the sample.

While assuming IRT MI to hold may be convenient from a theoretical and a practical perspective, this assumption may be too restrictive to be realistic in practice (Schmitt & Kuljanin, 2008; Vandenberg & Lance, 2000), and it can be violated in a variety of ways. Violations of MI at the level of individual items have received considerable attention in the literature, where a variety of methods for detecting differential item functioning (DIF; Mellenbergh, 1989) across observed groups has been proposed (Ackerman, 1992; Bock & Zimowski, 1997; Holland & Wainer, 1993). If there is DIF for a particular item, the parameters of the IRF of that item are taken to depend on group membership (e.g., gender or ethnicity).

A limitation of these standard multi-group approaches to investigating and modeling DIF is that they require membership of the relevant groups to be known. Mixture IRT (Rost, 1990) combines IRT modeling with latent class modeling, and provides a more general way of addressing a lack of MI than multi-group approaches, in the sense that the relevant grouping variable is no longer assumed to be manifest (Samuelsen, 2008). This makes mixture IRT a flexible and general approach that allows researchers to gain a deeper insight into the sources and nature of DIF, which is of great scientific and practical importance.

Numerous mixture IRT methods have been proposed (e.g., see Bolt, Cohen, & Wollack, 2001; Cho, De Boeck, Embretson, & Rabe-Hesketh, 2014; Cohen & Bolt, 2005; von Davier & Yamamoto, 2004; von Davier & Rost, 1995; Rost, 1990, 1991; Rost, Carstensen, & von Davier, 1997; Smit, Kelderman, & van der Flier, 2000). A framework that is particularly noteworthy because of its generality is the one proposed by von Davier (2008), which allows the user to consider different functional forms of the relationship between the manifest and the latent variable(s). However, the framework is still limited in the sense that across different mixture components the functional form is the same, for example with the model in all mixture components being a partial credit model. As a consequence, a form of IRT MI is still assumed: While *item parameters* are allowed to *differ* for the classes, the IRFs are still assumed to be of the *same parametric form* for all classes.

While notably less common, there has also been some interest in considering mixture models that do not have the same IRT model in all classes. An early example is the HYBRID model (von Davier, 1996; Yamamoto, 1987; 1989), which proposes a mixture of classes where some classes are scalable (i.e., a standard IRT model holds and the trait of interest is measured), while other classes are not (i.e., item probabilities do not depend on a latent trait). This model has for example been used to separate random responders on multiple choice tests from those who respond based on ability (Mislevy & Verhelst, 1990), and to model speededness in educational testing (Yamamoto & Everson, 1995). On the one hand, HYBRID models can be considered to have different structural models in each mixture component. On the other hand, as Von Davier and Yamamoto show (2007), HYBRID models can be represented as mixture models with mixture components that have the same parametric form, but with constraints imposed on some model parameters. For example, a HYBRID model with a latent class component and a Rasch model component can be seen as a mixture Rasch model in which in one class the variance of ability is constrained to be zero (von Davier & Yamamoto, 2007, p. 107). Other researchers have also suggested to consider IRT mixture models where in some components constraints are placed on some of the model parameters. A relevant example is the work by De Boeck, Cho, and Wilson (2011), who proposed a mixture IRT model for explaining DIF by introducing a secondary dimension that influences the response probabilities in only one of the two latent classes. Effectively, this results in a mixture model of two two-dimensional IRT models of the same parametric form, but where for all items the discrimination parameter for the second dimension is fixed to zero in the non-DIF class, while it is allowed to be nonzero for the DIF items in the DIF class. A similar approach was considered in the context of modeling cheating (Shu et al., 2013), where every cheater obtains a person-specific increase in ability, but only on items that were exposed (i.e., some items having nonzero loading on this extra dimension, but only for the group of cheaters). One could consider such models to present a mixture of structurally different measurement models, but only in the sense that the nested models differ in the number of freely estimated parameters. To our knowledge, existing mixture IRT approaches have all focused on sets of measurement models where any differences in the structure of these measurement models are due to some model parameters being fixed for some of the classes. As far as we know, no mixture IRT models have been proposed where the measurement models have a fundamentally different structure, in the sense that for the different classes the measurement models are not all (possibly constrained) versions of one general measurement model.

If there are important qualitative differences between the response processes in the different classes, the assumption of having the same or a similar measurement model for all classes may be unrealistic. For example, persons may differ in their response styles or strategies when answering survey questions (Baumgartner & Steenkamp, 2001), which may be difficult to incorporate using IRFs of the same parametric form. In general one can argue that it may not be realistic to assume that differences in the response processes in the different classes can be fully captured using a single type of measurement model rather than resulting in structurally different measurement models being appropriate for the different classes.

In this paper, we propose a general mixture IRT framework that allows for structurally different measurement models in different classes, while still keeping these models connected through the inclusion of a shared set of latent variables that (partially) explain the observed response patterns. The different measurement models do not need to be nested, nor do they have to be special cases of a more general measurement model. The approach proposed in this paper makes it possible to obtain information for all persons about the attributes intended to be measured, even if there are important qualitative differences across classes in the cognitive processes that relate these attributes to the responses. The approach requires the researcher to formulate competing measurement models that may hold for an unknown subsection of the population, after which a mixture model can be estimated that includes these different measurement models. In this framework, class membership and IRT person and item parameters can be estimated concurrently.

The structure of the remainder of the paper is the following. “Two measurement models for Likert-scale data” discusses an issue in the context of modeling Likert-scale data, where it is plausible that two structurally different measurement models are needed to account for different uses of the item categories across persons. In “Using the general mixture IRT approach: a two-class mixture model for Likert-scale data”, the proposed general mixture IRT framework is illustrated by considering the specification of a mixture model that makes use of the two measurement models discussed in “Two measurement models for Likert-scale data”, and a Bayesian estimation procedure is proposed. “Simulation study” evaluates the performance of this procedure under a variety of conditions using a simulation study, considering both classification accuracy and parameter recovery. Subsequently, the procedure is applied to an empirical example (“Empirical example”), to illustrate the possible gains from considering mixture models that incorporate structurally different measurement models. The paper concludes with a discussion that considers both the specified two-class mixture model for Likert scales and the proposed mixture IRT framework in general.

## Two measurement models for Likert-scale data

In many applications in the social sciences, attributes are measured using Likert scales (Likert, 1932; Cronbach, 1950). These scales consist of items that have multiple answer-category options, allowing respondents to select a category that they feel is most appropriate. Often, these Likert items ask respondents to indicate the extent to which they agree or disagree with a certain statement, using ordered categories that in some form or other are supposed to match a certain level of agreement. These responses are then coded into item scores, which are taken to be indicative of the attribute of interest, and which can be analyzed using a statistical model.

It is important to emphasize that while the coded responses result in numerical item scores, the response categories are qualitative in nature. Thus, it is not necessarily the case that the differences between an item score of 1 (e.g., “strongly disagree”) and 2 (e.g., “disagree”) in terms of the severity of the position are the same as the difference between an item score of 2 and 3 (e.g., “neither agree nor disagree”). Furthermore, due to the qualitative nature of the categories, there are also likely to be differences *across persons* in the way persons interpret and make use of these categories. This complicates the analysis of the response data using polytomous IRT methods, because it implies that different measurement models are appropriate for different persons.

Of particular relevance in this context is the middle category that is often present in Likert items, for example formulated as “neither agree nor disagree” or “neutral”. While including such a middle category gives respondents the possibility to communicate a neutral position towards the presented statement, respondents differ in their interpretation and use of this neutral category: Some respondents select the neutral category to indicate that their position falls somewhere in between the two adjacent categories (e.g., in between “agree” and “disagree”), but others treat the neutral category as a nonresponse option that indicates that they do not have (or do not want to communicate) an opinion regarding the statement that is presented (Kalton, Roberts, & Holt, 1980; Raaijmakers, Van Hoof, ’t Hart, Verbogt, & Vollebergh, 2000; Sturgis, Roberts, & Smith, 2014). While eliminating the middle category altogether may help avoid this issue, this prevents respondents from being able to communicate a neutral position on the item, which may be a valid response to the item (Presser & Schuman, 1980).

If the middle category is included, one can attempt to model possible between-person differences in response style with respect to the use of that category. Existing model-based approaches to dealing with response styles aim to model a person’s tendency to use the middle response category, which is often labeled ‘midpoint responding’ (Baumgartner & Steenkamp, 2001). In order to explain between-person differences in how often the middle category is used, in these approaches either an additional continuous latent variable is added to the model (e.g., see Böckenholt, 2012; Bolt, Lu, & Kim, 2014; Falk & Cai, 2016; Jeon & De Boeck, 2016; Khorramdel & von Davier, 2014; Tutz & Berger, 2016), or the use of different person mixture components is considered (e.g., see Hernández, Drasgow, & González-Romá, 2004; Maij-de Meij, Kelderman, & van der Flier, 2008; Moors, 2008; Rost et al., 1997). As a result persons are either placed somewhere on a dimension that captures the midpoint responding tendency^1^ seen as a continuous trait, or are placed in latent classes that differ in their propensities towards choosing the middle category.

In contrast, we propose to consider two qualitatively and fundamentally different ways in which respondents use the middle category. That is, we focus not on the between-person differences in *how often* the middle category is used, but in *how* this category is used: either as an ordered category located between “agree” and “disagree”, or as a nonresponse option. This constitutes a fundamental and qualitative difference in how persons use the categories. While researchers have been interested in capturing such qualitative differences between classes, approaches that have been suggested so far (e.g., see Hernández et al.,, 2004; Maij-de Meij et al., 2008; Moors, 2008; Rost et al.,, 1997) have relied on mixture IRT models that assume the same parametric form for all classes. However, we argue that current mixture IRT models are not optimally equipped to address this issue, as it is not mainly about *quantitative differences* that may exist in the way different persons use or interpret the scale (e.g., with persons differing in their interpretation of how strongly one has to agree with a statement before selecting “strongly agree”; Greenleaf, 1992; Jin & Wang, 2014), but rather about whether the person takes the middle category to belong to the scale at all. To appropriately deal with this, it may be necessary to consider a mixture of structurally different measurement models that addresses the qualitative differences that exist in the interpretation and use of the middle category, as will be discussed in the following sections.

### Qualitatively similar categories

The standard way of dealing with the middle category on Likert items is to assume that respondents who choose the middle category select this category to indicate a neutral level of endorsement, just as they would select the category “strongly agree” to indicate strong positive endorsement. This amounts to treating the categories as being quantitatively different (i.e., indicating different degrees of endorsement) but qualitatively similar (i.e., all of them being indicative of the degree of endorsement of the same statement and hence of the same attribute). Thus, the item scores are considered to be ordinal, and it may be appropriate to model these using polytomous IRT models. Let *X*_*pi*_ be the score of person *p* on item *i* which can take on values {1,2,…,*m*}, where the maximum score *m* is odd and $\frac {m + 1}{2}$ is the middle category. We will for notational convenience also assume that all item scores are ordered in accordance with the direction of the scale.

**Fig. 1:**
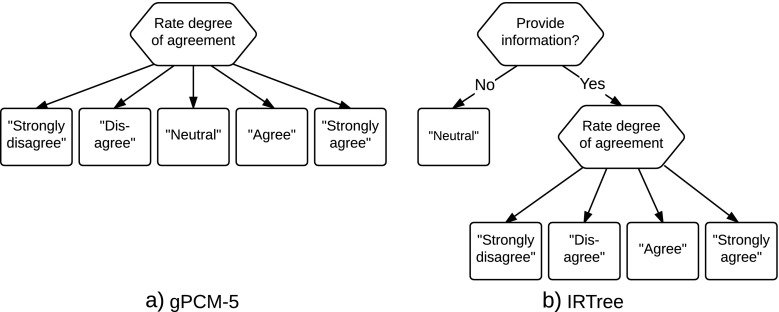
Two decision trees for a five-category Likert-scale item

Several polytomous IRT models exist that could be used to analyze Likert-type data, which differ in their specification of the IRF (Andrich, 1978; Bock, 1972; Masters, 1982; Muraki, 1992; Samejima, 1969). Because for this paper the aim is to illustrate that measurement models of different structures may be needed to optimally explain the item responses, we want to make use of a relatively flexible and unrestrictive IRT model, which is why we consider the generalized partial credit model (Muraki, 1992), which we here denote by gPCM-*m* to indicate that *m* categories are modeled. In the gPCM-*m*, item scores are modeled through

1 $$ g\left( X_{pi}\,|\, \theta_{pd_{i}},\alpha_{i},\boldsymbol{\delta}_{i}\right)= \frac{\exp\left( {\sum}_{k = 1}^{X_{pi}}\left( \alpha_{i}\left( \theta_{pd_{i}}-\delta_{ik}\right)\right)\right)} {{\sum}_{s = 1}^{m}\exp\left( {\sum}_{k = 1}^{s}\left( \alpha_{i}\left( \theta_{pd_{i}}-\delta_{ik}\right)\right)\right)},  $$

where $\theta _{pd_{i}}$ is the person parameter of person *p* on dimension *d*_*i*_ that item *i* is designed to capture, *α*_*i*_ is the slope of item *i*, and ***δ***_*i*_ = *δ*_*i*1_,…,*δ*_*im*_ is a vector of thresholds of the *m* categories, with *δ*_*i*1_ = 0 for identification. For each response the gPCM-*m* can be represented as a decision tree with one node and *m* possible outcomes (see Fig. 1a for *m* = 5).

It is rather common for questionnaires to consist of multiple Likert scales each designed to measure a single latent trait. Suppose a test is intended to measure *D* dimensions, such that for each person *p* the set of latent variables ***𝜃***_*p*_ = {*𝜃*_*p*1_,…,*𝜃*_*pD*_} is of interest. Let us by **d** = {*d*_1_,…,*d*_*K*_} denote a design vector specifying to which dimension each item belongs, where *d*_*i*_ = *d* indicates that item *i* belongs to dimension *d*. Since each item only captures one dimension, the item scores can be modeled through Eq. 1. When *D* = 1, the subscript *d*_*i*_ can be dropped and Eq. 1 becomes the unidimensional gPCM (Muraki, 1992).

### Qualitatively different categories

Using a gPCM (or a mixture of gPCMs) may be appropriate if respondents consider the categories to differ only quantitatively, meaning that they take each category to reflect a particular degree of endorsement and hence that we can treat the item scores as ordinal. However, this would not be defensible if respondents interpret the middle response category as being qualitatively different from the other categories, for example by approaching it as a noninformative response option. In that case, standard polytomous IRT models are not appropriate for modeling the item scores, because a respondent’s choosing the middle category cannot be explained by an appeal to the dimension that the item is supposed to capture.

Instead, it may be possible to model the response process through the use of IRTree models (Böckenholt, 2012; De Boeck & Partchev, 2012; Jeon & De Boeck, 2016). Using IRTrees, one can model a response process that contains multiple steps through the use of nodes, with each possible response option corresponding to one specific path through the IRTree. Each node in the IRTree corresponds to a specific statistical model, which may differ for different nodes. Importantly, nodes within a single IRTree model can differ with respect to the latent variables that play a role in them.

To capture the response process where the middle category of a Likert-scale item is taken to represent a nonresponse option, we propose to model the responses with an IRTree model with two nodes, in line with the IRTree models discussed by Jeon and de Boeck (2016). For *m* = 5 this IRTree model is illustrated in Fig. 1b. The first node represents whether the person chooses the middle category (i.e., decides to avoid giving an informative response to the question) or not. We assume here that whether or not an informative response is given will depend both on the item that is answered and the person that answers the item. That is, as is common in the response-style literature, we assume that persons differ in the degree to which they will display a response style (e.g., see Böckenholt, 2012; Tutz & Berger, 2016) and this response style is assumed to be stable (i.e., constitutes a person trait; e.g., see Baumgartner & Steenkamp, 2001; Greenleaf, 1992). Following this assumption, we propose to consider a single latent variable that captures between-person differences in the usage of the middle category on the items on the test. This latent variable can provisionally be thought of as corresponding to a trait that captures a person’s tendency to avoid giving informative responses. The second node of the IRTree comes into play only if the middle category is not chosen. In this case the respondent chooses from the *m* − 1 remaining categories and the choice depends on the latent trait that the scale was designed to measure.

To apply the IRTree model, we need to re-code the item score *X*_*pi*_ into two different variables (see also De Boeck & Partchev, 2012). For the first node, we recode *X*_*pi*_ into a dichotomous outcome variable $X_{pi}^{*}$ indicating whether the middle category was selected (*Xpi*∗ = 1 ) or not (*Xpi*∗ = 0 ). For the second node, we recode *X*_*pi*_ into an ordinal outcome variable *Xpi*∗∗ = 1,…,*m* − 1, such that

2 $$ X_{pi}^{**}=X_{pi}-\mathcal{I}\left( X_{pi}>\frac{m + 1}{2}\right).  $$

If $X^{*}_{pi}= 1$ the response process has terminated after the first node and hence $X_{pi}^{**}$ is missing.

Both nodes of the IRTree can be modeled using common IRT models. In line with Jeon and De Boeck 2016, we propose to model the first node of the IRTree using a two-parameter logistic model (2PL; Lord & Novick, 1968):

3 $$ h_{1}\left( X^{*}_{pi}\,|\, \theta_{p0},\alpha_{iS},\delta_{iS}\right)= \frac{\left( \exp\left( \alpha_{iS}\left( \theta_{p0}-\delta_{iS}\right)\right) \right)^{X_{pi}^{*}}}{1+\exp\left( \alpha_{iS}\left( \theta_{p0}-\delta_{iS}\right)\right)},  $$

where *𝜃*_*p*0_ denotes the person parameter of person *p*, *α*_*iS*_ and *δ*_*iS*_ denote the slope and the location parameter of item *i*, and the subscript *S* refers to “**S** kipping” giving an informative response. Here, *𝜃*_*p*0_ is a latent variable that captures a respondent’s tendency to skip items by choosing the nonresponse option, with low values of *𝜃*_*p*0_ indicating a relatively strong tendency to provide informative answers (i.e., to avoid using the middle category). It can be emphasized here that the model in Eq. 3 assumes the existence of both person- and item-level effects: Persons are taken to differ in their overall tendency to select the middle category (*𝜃*_*p*0_), but items also differ in the extent to which people are inclined to provide noninformative responses to it (i.e., the item location *δ*_*iS*_ can vary across items). This is in line with the idea that item content and formulation can play an important role in determining the extent to which nonresponse occurs, but that the probability of observing a noninformative response will also depend on person characteristics such as response style.

The second node of the IRTree captures the selection of a response category relevant for the attribute of interest, for which we propose to use the gPCM. Thus, a respondent’s choice for one of the (*m* − 1) remaining categories (i.e., after eliminating the middle category) is modeled through

4 $$ h_{2}(X^{**}_{pi}\,|\,\theta_{pd_{i}},\alpha_{iT},\boldsymbol{\delta}_{iT})= \frac{\exp\left( {\sum}_{k = 1}^{X^{**}_{pi}} \alpha_{iT}\left( \theta_{pd_{i}}- \delta_{ikT}\right)\right)} {{\sum}_{s = 1}^{m-1}\exp\left( {\sum}_{k = 1}^{s}\alpha_{iT}\left( \theta_{pd_{i}}-\delta_{ikT}\right)\right)},  $$

where *α*_*iT*_ and ***δ***_*iT*_ denote the slope and the threshold parameters of item *i*, and subscript *T* refers to the “IR**T** ree” in order to differentiate these parameters from those in the gPCM-*m*.

To model *X*_*pi*_, the models at the two nodes can be combined to obtain

5 $$\begin{array}{@{}rcl@{}} &&h\left( X_{pi}\,|\,\theta_{p0},\theta_{pd_{i}},\alpha_{iS},\delta_{iS},\alpha_{iT},\boldsymbol{\delta}_{iT}\right) \\ &=& h_{1}\left( X^{*}_{pi}\,|\, \theta_{p0},\alpha_{iS},\delta_{iS}\right)\left( h_{2}(X^{**}_{pi}\,|\,\theta_{pd_{i}},\alpha_{iT},\boldsymbol{\delta}_{iT})\right)^{1-X^{*}_{pi}}. \end{array} $$

The differences in structure between the IRTree and the gPCM-*m* can be observed by contrasting Eq. 5 with Eq. 1, where it may also be noted that the gPCM-*m* is not a special case of the IRTree model in Eq. 5. To determine which of the two models should be preferred, one could compare their fit to the data. However, if one part of the population treats the middle category as a noninformative response option while the other part of the population responds in line with the gPCM-*m*, neither model will fit the data very well, and using either one of them would result in estimates for the person and item parameters that will to some degree be biased. In such cases, it may be preferable to consider a mixture of the two models, as will be discussed in the next section.

## Using the general mixture IRT approach: a two-class mixture model for Likert-scale data

When researchers suspect that IRT MI is violated and that classes of persons exist that differ qualitatively in their response processes, they can consider making use of the general mixture IRT approach proposed in this paper. This requires the researcher to formulate different measurement models that capture the suspected differences in the underlying response processes. One constraint that can be imposed is that the different measurement models are connected through the inclusion of a shared set of relevant latent variables. The scales for the different classes can then be linked by imposing constraints either on the person-side of the model (identical distribution of these latent variables in all classes) or on the item-side (if parameters with a similar function are present in all classes; discussed for regular mixture IRT models in Paek & Cho, 2015).

In the context of modeling Likert-scale data, one can consider using a mixture of the two models that have been proposed in the previous section. Here, we propose to consider a person mixture. That is, we assume that persons belong to one of the two classes, and that this class membership is fixed throughout the test. Thus, class membership is taken to be a person property, and persons are assumed to stick to one interpretation of the middle response category for all the items on the test. To connect the two models, one can make the assumption that the latent variables ***𝜃***_*p*_ (i.e., excluding *𝜃*_0_) that play a role in the second node of the IRTree model are the same as those present in the gPCM-*m* (see also Fig. 1). For this particular mixture model we propose to link the scales by assuming the same distribution of the latent trait(s) in both classes.

For this two-class mixture model for Likert-scale data, the probability of a certain item score for person *p* on item *i* depends on the class membership of person *p*, denoted by *Z*_*p*_. *Z*_*p*_ = 1 if the person belongs to the gPCM-*m* class, and *Z*_*p*_ = 0 if the person belongs to the IRTree class. The response of person *p* to item *i* can be modeled as:

6 $$\begin{array}{@{}rcl@{}} &&f(X_{pi}\,|\, \theta_{p0},\theta_{pd_{i}},\alpha_{iR},\boldsymbol{\delta}_{iR},\alpha_{iS},\delta_{iS},\alpha_{iT},\boldsymbol{\delta}_{iT},Z_{p})\\ &=& \left( g(X_{pi} \,|\, \theta_{pd_{i}},\alpha_{iR},\boldsymbol{\delta}_{iR})\right)^{Z_{p}}\times \left( \vphantom{{\sum}_{1}}h_{1}\left( X^{*}_{pi}\,|\, \theta_{p0},\alpha_{iS},\delta_{iS}\right)\right.\\ &&\times\left.\left( h_{2}(X^{**}_{pi}\,|\,\theta_{pd_{i}},\alpha_{iT},\boldsymbol{\delta}_{iT})\right)^{1-X^{*}_{pi}} \vphantom{{\sum}_{1}}\right)^{1-Z_{p}} \end{array} $$

where *α*_*iR*_ and *δ*_*ikR*_ are used instead of *α*_*i*_ and *δ*_*ik*_ for the slope and the threshold parameters in the gPCM-*m* to unify the notation.^2^ To estimate this mixture model, a Bayesian MCMC algorithm can be employed. The specification of the prior distributions and the estimation procedure is the topic of the next two subsections.

### Prior distributions

For each person *p*{*𝜃*_*p*0_,*𝜃*_*p*1_,…,*𝜃*_*pD*_} are assumed to have a multivariate normal distribution with a zero mean vector and a (*D* + 1) × (*D* + 1) covariance matrix **Σ**. The mean is constrained to 0 for identification, because in IRT models only the difference (*𝜃* − *δ*) is identified and not the parameters themselves (Hambleton, Swaminathan, & Rogers, 1991). The variances in **Σ** are also not identified, however to simplify the conditional posterior distributions and to improve convergence instead of constraining them we estimate **Σ** freely, and at each iteration of the Gibbs Sampler re-scale the parameters such that all the variances in **Σ** are equal to 1.

For the hyper-prior of **Σ** we choose an inverse-Wishart distribution with *D* + 3 degrees of freedom and **I**_*D*+ 1_ as the scale parameter. With this choice for the prior degrees of freedom, the posterior is not sensitive to the choice of the prior scale parameter, because in the posterior distribution the prior is dominated by the data when *N* ≫ *D* + 3 (Hoff, 2009, p. 110).

All *Z*_*p*_s are assumed to have a common prior Bernoulli distribution with the hyper-parameter *π* specifying the probability of a person randomly drawn from the population belonging to the gPCM-*m* class. This is a hierarchical prior (Gelman, Carlin, Stern, & Rubin, 2014), that is, for each person the posterior class probability depends on the proportion of persons in this class. This results in shrinkage of the estimates of persons’ class memberships: If one of the classes is small, then the proportion of persons estimated to belong to this class would be even smaller. The advantage of this prior is that if one of the classes is absent this class will most likely be estimated to be empty, which would often not happen if instead an independent uniform prior would be used for each person. As the prior of *π* we use $\mathcal {B}(1,1)$, such that a priori all values between 0 and 1 are taken to be equally likely.

A priori, the item parameters are assumed to be independent of each other. For each of the item slope parameters (*α*_*iR*_,*α*_*iS*_,*α*_*iT*_), a log-normal prior distribution (to ensure these parameters to be positive) with a mean of 0 and variance of 4 is used. Using a relatively large variance compared to the range of values that the logs of slope parameters normally take on ensures that the prior is relatively uninformative and that the posterior will be dominated by the data (Harwell & Baker, 1991). The following prior is used for the item threshold and location parameters:

7 $$\begin{array}{@{}rcl@{}} p(\boldsymbol{\delta}_{iR},\delta_{iS},\boldsymbol{\delta}_{iT})\!\propto\! \mathcal{N}\!\left( \delta_{iS}; 0,10 \right)\mathcal{I}(\delta_{i1R}= 0)\mathcal{I}(\delta_{i1T}= 0)\!\prod\limits_{k = 2}^{m}\! \mathcal{N}\!\left( \delta_{ikR}; 0,10 \right)\!\prod\limits_{k = 2}^{m-1}\! \mathcal{N}\!\left( \delta_{ikT}; 0,10 \right). \end{array} $$

Here, large variances are again used for the parameters to ensure that the prior is relatively uninformative. As has been mentioned before, *δ*_*i*1*T*_ = *δ*_*i*1*R*_ = 0 for identification.

### Estimation

The model can be estimated by sampling from the joint posterior distribution of the model parameters:

8 $$\begin{array}{@{}rcl@{}} p(\boldsymbol{\theta}_{0},\boldsymbol{\theta},\boldsymbol{\alpha}_{T},\boldsymbol{\delta}_{T},\boldsymbol{\alpha}_{S},\boldsymbol{\delta}_{S}, \boldsymbol{\alpha}_{R},\boldsymbol{\delta}_{R},\mathbf{Z},\boldsymbol{\Sigma},\pi\,|\, \mathbf{X})\propto p(\boldsymbol{\Sigma}) p(\pi) \prod\limits_{p} \left( p(\theta_{p0},\boldsymbol{\theta}_{p}\,|\, \boldsymbol{\Sigma})p(Z_{p}\,|\, \pi)\right)\\ \times\prod\limits_{i} p(\alpha_{iS},\boldsymbol{\delta}_{iS},\alpha_{iS},\delta_{iS},\alpha_{iR},\boldsymbol{\delta}_{iR}) \prod\limits_{p}\prod\limits_{i} f(X_{pi}\,|\, \theta_{p0},\theta_{pd_{i}},\alpha_{iR},\boldsymbol{\delta}_{iR},\alpha_{iS},\delta_{iS},\alpha_{iT},\boldsymbol{\delta}_{iT},Z_{p}), \end{array} $$

where ***𝜃***_0_ is a vector of *𝜃*_*p*0_s of all persons and ***𝜃*** is an *N* × *D* matrix of person parameters of all persons on all dimensions 1 to *D*; ***α***_*T*_,***α***_*S*_, and ***α***_*R*_ are the vectors of *α*_*iT*_s, *α*_*iS*_s, and *α*_*iR*_s of all the items, respectively; ***δ***_*T*_ and ***δ***_*R*_ are the matrices of threshold parameters of all the items in the gPCM-*m* and gPCM-(*m* − 1), respectively; ***δ***_*S*_ is a vector of *δ*_*i*_s of all the items; **Z** is a vector *Z*_*p*_s of all persons. To sample from the posterior distribution in Eq. 8 we developed a Gibbs Sampler algorithm (Geman & Geman, 1984; Casella & George, 1992) in R (R Core Team, 2015). The Appendix contains the description of the algorithm, and the code is available in the Online Supplementary Materials.

To start the Gibbs Sampler, starting values for the model parameters need to be specified (see Appendix for the details). To remove the effect of the starting values on the results, the first part of the sampled values (i.e., burn-in) is removed. Even after discarding the burn-in, the results of the algorithm might still depend on the starting values of **Z** when a finite number of iterations are used for the burn-in period. For example, if at the start of the algorithm none of the persons who belong to the IRTree are assigned to the IRTree class, then the IRTree item parameters will not be sampled optimally and it is possible that the class will initially become empty, because the non-optimized IRTree model will not fit the data of these persons better than the gPCM-*m*. To avoid ending up with a chain stuck in such a local maximum (i.e., having an empty class even though that class should not be empty), we recommend the use of multiple chains (Gamerman & Lopes, 2006), retaining the results of the best chain chosen based on the average post-burn-in log-likelihood:

9 $$ L_{c}=\frac{1}{T}\sum\limits_{t} \sum\limits_{p}\sum\limits_{i} \ln f(X_{pi}\,|\,\theta^{tc}_{p0},\boldsymbol{\theta}^{tc}_{p},\alpha^{tc}_{iT}, \boldsymbol{\delta}^{tc}_{iT},\alpha^{tc}_{iS},\delta^{tc}_{iS},\alpha^{tc}_{iR},\boldsymbol{\delta}^{tc}_{iR},Z^{tc}_{p}),  $$

where *L*_*c*_ is the average post-burn-in log-likelihood in chain *c*, the superscripts *t* and *c* denote the values of a parameter in the *t*-th post-burn-in iteration in the *c*-th chain, and *T* denotes the number of post-burn-in iterations. By using a diverse set of starting values and retaining the chain for which *L*_*c*_ is highest, the risk of obtaining a solution based on a local maximum can practically be avoided (Gamerman & Lopes, 2006).

The sampled values of the parameters from all post-burn-in iterations in the best chain are used to approximate the joint posterior distribution in Eq. 8, which can be used to obtain estimates of the parameters. This approach automatically takes the uncertainty about the class membership of persons into account in the posterior of ***𝜃***, as the marginal posterior of ***𝜃***_***p***_ is a weighted mixture of the two posteriors of ***𝜃***_***p***_ conditional on the class membership:

10 $$\begin{array}{@{}rcl@{}} p(\boldsymbol{\theta}_{\boldsymbol{p}}) &=& p(\boldsymbol{\theta}_{\boldsymbol{p}}|Z_{p} = 0)p(Z_{p} = 0) \\ &&+ p(\boldsymbol{\theta}_{\boldsymbol{p}}|Z_{p} = 1)p(Z_{p} = 1). \end{array} $$

For all continuous parameters, we use the corresponding posterior means as their estimates, which are approximated by the averages of the corresponding post-burn-in sampled values. For each *Z*_*p*_ the posterior mode is used as an estimate. The posterior probability of a person belonging to a certain class is approximated by the proportion of iterations in which this person has been assigned to this class.^3^

## Simulation study

While the proposed general mixture IRT framework allows researchers to specify structurally different measurement models, the practical usefulness of such an approach will depend on the extent to which the different measurement models can be successfully distinguished in realistic sets of data with a limited amount of information available per person and per item. For measurement to improve through the use of these mixture models it is crucial that persons can be classified with a high degree of accuracy and that parameters can be recovered. The extent to which this is possible will depend on the particular measurement models that are considered, which makes a general assessment of the feasibility of using the proposed approach in practice difficult. However, if the procedure can be used successfully under realistic conditions in the context of the proposed two-class mixture model for Likert-scale data, this may inspire confidence that application of the approach in other contexts is feasible and useful as well. To assess the range of conditions within which the proposed procedure does and does not show acceptable performance, a simulation study was performed that assessed classification accuracy (“Classification accuracy”). To assess the extent to which item parameters are estimated correctly and the extent to which using the mixture model improves the accuracy of person estimates compared to using a nonmixture gPCM when two different classes are present, a small-scale follow-up simulation study was also performed that considers recovery of the item parameters of the mixture model and compares the recovery of persons’ latent trait values under the two models (“Parameter recovery”).

### Classification accuracy

#### Method

##### Design

Four design factors were considered: sample size (*N* = 500,1000,2000), number of items (*K* = 20,40), number of dimensions that the test is intended to measure (*D* = 1,2; items distributed equally for *D* = 2), and proportion of persons belonging to the IRTree class (*P* = 0,.25,.5). For the simulation study a full factorial 3 × 2 × 2 × 3 design was used. Five-point Likert scales were considered with persons either belonging to the gPCM-5 class or to the IRTree class with the 2PL in the first node and the gPCM-4 in the second node. In each condition, 50 replicated data sets were generated using Eq. 6. In each replication, the model was estimated using the Gibbs Sampler with ten chains with 2000 iterations each (including 1000 iterations of burn-in; number based on pilot studies).

##### Parameter specification

For each condition the item and the person parameters were generated in the same way. For the first *N* × *P* persons in the sample *Z*_*p*_ = 0 (i.e., IRTree class) and the remaining *Z*_*p*_s were set to one (i.e., gPCM-5). All *𝜃* s were sampled independently from $\mathcal {N}(0,1)$. Thus, all person parameters were independent, matching the expectation that in most cases a response tendency would be orthogonal to the traits of interest.

Because the process of giving an informative response is assumed to be relatively similar across the two classes, the item parameters of the gPCM-5 and gPCM-4 were set to be correlated. The logs of *α*_*iT*_ and *α*_*iR*_ were sampled from a bivariate normal distribution with means equal to 0, variances equal to 0.25 and correlation of .5. Here a moderate correlation was chosen, as it accommodates the fact that having less categories available to provide an informative response may alter the discriminative properties of the item to some degree, while still being relatively similar under both models. The threshold parameters were sampled through $\boldsymbol {\delta }_{iR}= \bar \delta _{i} + \{-\bar \delta _{i},-1.5,-0.5,0.5,1.5\}$ and $\boldsymbol {\delta }_{iT}= \bar \delta _{i} + \{-\bar \delta _{i},-1.5,0,1.5\}$, where $\bar \delta _{i}\sim \mathcal {N}(0,1)$. This specification was chosen such that overall item locations, $\bar \delta _{i}$s, under the two models were the same, capturing the idea that having fewer categories should not alter the overall location of the item on the scale. For the first node of the IRTree, *α*_*iS*_ ∼*LogNorm*(− 0.5,0.25) and $\delta _{iS}\sim \mathcal {N}(2,0.25)$, which results in approximately 18% of responses in the IRTree class corresponding to the middle category. The low value of − 0.5 for the mean of ln*α*_*iS*_ was chosen to match the fact that items were not designed to measure a tendency to avoid giving informative responses.

##### Outcome measures

Under each condition the accuracy of the classification of the persons in the two classes was investigated. A person *p* was considered to be correctly classified if true class membership was equal to the estimate of *Z*_*p*_. For *P* = .25 and *P* = .5 several outcome variables were considered. *P*_*all*_ is the proportion of overall correctly classified persons, while *P*_*Tree*_ and *P*_*PCM*_ are the proportions of correctly classified persons among those whose true class membership is IRTree and gPCM-5, respectively. *P*_*cert*_ is the proportion of persons assigned to the correct class with high certainty (i.e., posterior probability of at least .95). For these four outcome measures, the average and standard deviation across the 50 replications were considered. For *P* = 0, the only outcome measure was the proportion of replications in which the IRTree class was estimated to be empty (i.e., all persons classified correctly).

#### Results

##### Overall results

The results of the simulation study are displayed in Table 1. The results obtained for *P* = 0 were very similar across all conditions, and are for that reason not displayed in Table 1. For *P* = 0, the IRTree class is consistently estimated to be empty: Across all replications in all conditions, it only happened once that the IRTree class did not become empty (for *N* = 2000, *K* = 40 and *D* = 1), and in that one replication only two persons out of 2000 were assigned to the IRTree class. Thus, there does not appear to be a risk of overfitting, meaning that empty classes are consistently identified as such.

**Table 1:** Results of the simulation study on classification accuracy

*N*	*K*	*D*	*P*		*P*_*all*_(SD)	*P*_*Tree*_ (SD)	*P*_*PCM*_ (SD)	*P*_*cert*_ (SD)
500	20	1	.25		.83 (.06)	.39 (.29)	.98 (.02)	.60 (.19)
			.5		.83 (.05)	.83 (.10)	.84 (.07)	.49 (.08)
		2	.25		.81 (.06)	.28 (.29)	.98 (.02)	.58 (.21)
			.5		.81 (.05)	.83 (.08)	.80 (.09)	.46 (.09)
	40	1	.25		.94 (.03)	.82 (.10)	.98 (.01)	.85 (.04)
			.5		.94 (.02)	.94 (.02)	.94 (.03)	.79 (.05)
		2	.25		.93 (.03)	.78 (.13)	.98 (.01)	.84 (.04)
			.5		.94 (.02)	.94 (.02)	.93 (.02)	.79 (.05)
1000	20	1	.25		.88 (.04)	.64 (.18)	.96 (.01)	.64 (.08)
			.5		.85 (.04)	.86 (.04)	.85 (.06)	.47 (.10)
		2	.25		.87 (.02)	.61 (.11)	.96 (.02)	.61 (.06)
			.5		.85 (.03)	.85 (.04)	.85 (.04)	.46 (.07)
	40	1	.25		.96 (.01)	.88 (.04)	.98 (.01)	.85 (.03)
			.5		.95 (.01)	.95 (.02)	.95 (.02)	.81 (.05)
		2	.25		.95 (.01)	.87 (.04)	.98 (.01)	.84 (.03)
			.5		.95 (.01)	.94 (.02)	.95 (.02)	.80 (.05)
2000	20	1	.25		.90 (.02)	.71 (.09)	.96 (.01)	.59 (.07)
			.5		.87 (.03)	.86 (.03)	.88 (.03)	.48 (.08)
		2	.25		.89 (.02)	.71 (.07)	.96 (.01)	.59 (.07)
			.5		.86 (.02)	.86 (.03)	.87 (.03)	.45 (.07)
	40	1	.25		.96 (.01)	.90 (.03)	.98 (.01)	.85 (.04)
			.5		.95 (.01)	.95 (.02)	.96 (.01)	.81 (.04)
		2	.25		.96 (.01)	.89 (.03)	.98 (.01)	.84 (.03)
			.5		.95 (.01)	.95 (.02)	.96 (.01)	.80 (.04)

Average values of *P*_*all*_ (overall proportion of correctly classified persons), *P*_*Tree*_ (proportion of correctly classified persons among those whose true class membership is IRTree), *P*_*PCM*_ (proportion of correctly classified persons among those whose true class membership is gPCM-5), and *P*_*cert*_ (proportion of persons that were assigned to the correct class with high certainty) and their standard deviations (SD) across 50 replications for different sample sizes (*N*), number of items (*K*), number of dimensions (*D*), and true proportions of persons belonging to the IRTree class (*P*)

In the majority of conditions the classification accuracy is encouraging: In all conditions the overall proportion of correctly classified persons (*P*_*all*_) exceeded .80 and in many cases .90 (see Table 1). The impact of the design factors on the outcome measures is discussed below. Because the results in Table 1 did not indicate any notable effect of the number of dimensions on classification accuracy, the subsequent discussion will focus on *D* = 1.

##### Sample size

As can be observed in Table 1, for most conditions sample size only had a small positive effect or even no clear effect on classification accuracy. However, sample size did have a notable impact on the proportion of persons correctly placed in the IRTree class (*P*_*Tree*_) when *K* = 20 and *P* = .25. Here, little information is available per person (because *K* = 20) and for *N* = 500 there is also little information available for estimating the IRTree item parameters (because only 125 persons belong to that class). Using the mixture model in this challenging condition may not be ideal, as the IRTree class was estimated to be empty in about 25% of replications, and the average *P*_*Tree*_ was low and its variance was high. For *K* = 20 and *P* = .25, with larger *N* the issue of the empty IRTree class disappeared, the average *P*_*Tree*_ improved, and its variance decreased.

##### Number of items

For all outcome measures, results improved markedly when increasing *K* from 20 to 40. The overall proportion of misclassified persons (1 − *P*_*all*_) is more than halved by this increase in test length, with *P*_*all*_ exceeding .90 in all conditions (see Table 1). Additionally, for *K* = 40 in all conditions approximately 80% or more of the classifications were both correct and made with the posterior probability of at least .95. This is a strong improvement over the conditions with *K* = 20, where *P*_*cert*_ was close to .5.

##### Class proportions

When both classes are present (i.e., *P* = .25 or *P* = .5), the relative size of the two classes did not appear to affect the overall proportion of correct classifications. However, the class proportions did have a strong impact on the classification accuracy for persons belonging to the IRTree. For *P* = .25, relatively few persons belong to the IRTree class. As will be further discussed in the next section, this complicates the estimation of the item parameters for that class. Additionally, because a hierarchical prior was used, the procedure’s posterior probability of a person belonging to a class depends on the estimated proportion of persons belonging to that class (Gelman et al., 2014). As a consequence, classification accuracy is likely to be reduced for the smaller class (but improved for the larger class). Because persons not assigned to the IRTree class are assigned to the gPCM class, *P*_*PCM*_ is larger when *P* = .25 compared to when *P* = .5.

### Parameter recovery

#### Method

To assess whether using the mixture model may improve measurement, the accuracy and precision of the estimates of both *𝜃*_1_ and the item parameters were investigated for the situation where *N* = 1000, *K* = 40 and *D* = 1. On the item side, we investigated how well the different item parameters were recovered. For the assessment of the recovery of the item location, we examined parameter recovery of the intercepts of the item and category characteristic functions (*β*_*iS*_ = −*α*_*iS*_*δ*_*iS*_, *β*_*ikT*_ = −*α*_*iT*_*δ*_*ikT*_, and *β*_*ikR*_ = −*α*_*iR*_*δ*_*ikR*_) rather than the location and threshold parameters, because the former were considered in the estimation procedure (see Appendix) as their estimates are more stable (see also Fox, 2010). Hence, investigating the recovery of the item and category intercepts (*β*_*iS*_,*β*_*ikT*_, and *β*_*ikR*_) provides a better insight into the degree to which the item location is correctly recovered by the procedure. On the person side, we investigated how well *𝜃*_1_ was recovered, and compared this with the recovery of *𝜃*_1_ under a nonmixture gPCM-5.

Three conditions were considered, which differed with respect to the proportion of persons belonging to the IRTree class (*P* = 0,.25,.5). A single set of item parameters and continuous person parameters was generated (see “Method”) and used for all three conditions. For each condition 100 data sets were simulated, for which the two models were estimated.^4^ The bias, variance, and mean squared error (MSE) of the estimates of the item parameters of the mixture model and of the estimates of *𝜃*_1_ under both models were investigated.

#### Results for the recovery of the item parameters

The item parameter recovery results are presented in Table 2. For *P* = 0, only the recovery of the gPCM-5 model is considered as in that condition the data do not contain information about the IRTree parameters. The results show that the average absolute bias is rather small for each type of parameter. Bias seems to be most notable for gPCM-4 parameters in the condition where *P* = .25, when there are relatively few persons in that class (0.134 and 0.132 for *α*_*iT*_ and *β*_*ikT*_, respectively). The average absolute bias in these parameters of the gPCM-4 appears to be approximately halved when the number of persons belonging to that class increases from 250 to 500 (*P* = .5). The only exception seems to be the intercept in the first node of the IRTree (*β*_*iS*_), where bias is low in both conditions.

**Table 2:** Average absolute bias (Bias), variance, and mean squared error (MSE) of the estimates of each type of item parameter in the mixture IRT model (1000 persons, 40 items, single dimension of primary interest; based on 100 replications)

		*P* = 0				*P* = .25				*P* = .5		
		Bias	Variance	MSE		Bias	Variance	MSE		Bias	Variance	MSE
*α*_*iR*_		0.027	0.010	0.011		0.010	0.014	0.014		0.015	0.025	0.025
*β*_*ikR*_		0.045	0.044	0.048		0.056	0.075	0.080		0.044	0.121	0.128
*α*_*iS*_		–	–	–		0.068	0.115	0.121		0.025	0.052	0.052
*β*_*iS*_		–	–	–		0.017	0.067	0.067		0.033	0.033	0.034
*α*_*iT*_		–	–	–		0.134	0.130	0.164		0.066	0.052	0.063
*β*_*ikT*_		–	–	–		0.132	0.531	0.595		0.065	0.218	0.236

With respect to the variance of the estimates, the item category intercept of the IRTree (*β*_*ikT*_) appears to be the most difficult to recover, especially when *P* = .25. The variance of the IRTree item parameter estimates is more than halved when class size is doubled (*P* = .5). Similarly, the variance of the estimates of the gPCM-5 parameters is also lowest when the gPCM-5 class is largest (*P* = 0) and gets worse when *P* > 0.

As the bias in the parameter estimates is small compared to their variance, the patterns observed for the MSE largely match those that were found for the variance. Thus, the MSE results indicate that of all parameters considered *β*_*ikT*_ is the most difficult to recover when class size is small, but that for all parameters the recovery greatly improves if the number of persons belonging to the relevant class increases. All of this suggests that class size strongly influences item parameter recovery, and that care should be taken to ensure that both classes have sufficient observations if the mixture model is to be used. When class size is reasonable (e.g., at least 500 persons in each class), item recovery of all relevant parameters appears to be adequate.

#### Results for the recovery of *𝜃*_1_

**Fig. 2:**
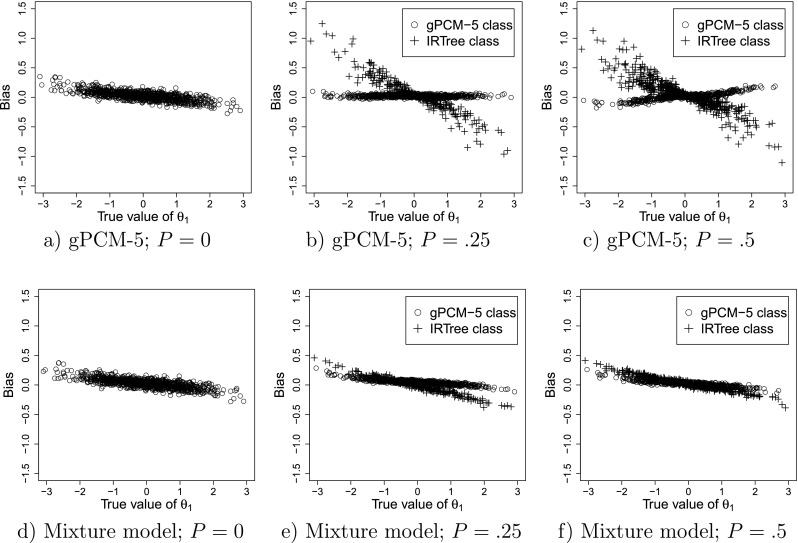
Bias of the estimates of *𝜃*_1_ under the nonmixture gPCM-5 (**a**, **b**, **c**) and the mixture model (**d**, **e**, **f**) when the true model is the mixture model with the true proportion of persons in the IRTree class equal to *P*. Each *point* represents a single person

**Fig. 3:**
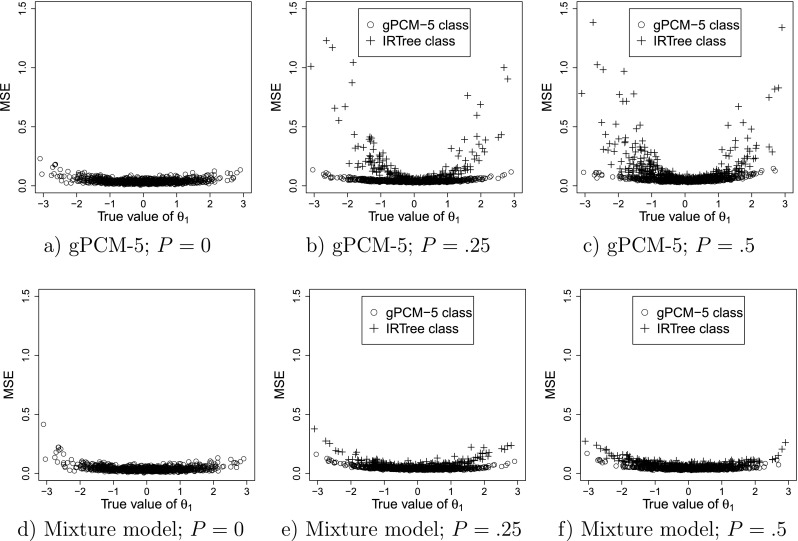
Mean squared error (MSE) of the estimates of *𝜃*_1_ under the nonmixture gPCM-5 (**a**, **b**, **c**) and the mixture model (**d**, **e**, **f**) when the true model is the mixture model with the true proportion of persons in the IRTree class equal to *P*. Each *point* represents a single person

The results for the recovery of *𝜃*_1_ are displayed in Fig. 2, which provides a graphical display of the bias of the estimates of *𝜃*_1_ observed under both the gPCM-5 and the mixture model. The MSEs for these two models are displayed in Fig. 3.

##### Empty IRTree class

The results of the gPCM-5 and the mixture model are practically identical when *P* = 0 (see Figs. 2 and 3). This indicates that using the mixture model when in fact using only the gPCM-5 would have sufficed does not deteriorate the quality of the estimates of *𝜃*_1_. For this condition, the average absolute bias of the estimates of *𝜃*_1_ in both models was 0.06. The average variance of the estimates was 0.03, and the average MSE was 0.04 for both models. As can be seen in Fig. 3a and d, the MSEs of the estimates increase when moving away from 0. In this condition, low *𝜃*_1_s are overestimated while high *𝜃*_1_s are underestimated, as illustrated in Fig. 2a and d. This shrinkage towards the mean is in both models due to the use of a hierarchical model (Fox, 2010). Such shrinkage can be considered desirable because it minimizes prediction error, therefore one would ideally like to observe the same amount of shrinkage in the other conditions (i.e., when *P* > 0). Deviations from the pattern observed for *P* = 0 can be taken to indicate lack of robustness of the model inferences for *P* > 0, that is, that estimates of *𝜃*_1_ differ from those that would have been obtained if all persons had belonged to the gPCM-5 group.

##### Small IRTree class

For *P* = .25, the average absolute bias and the average MSE was slightly lower for the mixture model (0.07 and 0.05, respectively) than for the gPCM-5 model (0.08 and 0.07, respectively), while the average variance was similar (0.045 for both models). As can be seen in Fig. 2b, under the nonmixture gPCM-5 the *𝜃*_1_ of persons belonging to the IRTree class is highly overestimated at the lower end and underestimated on the higher end of the *𝜃*_1_-scale, resulting in a relatively large average absolute bias and MSE for this group (0.24 and 0.17, respectively). For persons with high or low true values of *𝜃*_1_ and belonging to the IRTree class the MSEs were much lower when the mixture model was used (Fig. 3e) than when the nonmixture gPCM-5 was used (Fig. 3b).

When using the nonmixture gPCM-5, the parameters of the persons whose true class is the gPCM-5 were only slightly biased (0.03), and while there is still shrinkage to the mean for this group, the observed effect was smaller than for *P* = 0. In the mixture model for persons belonging to the gPCM-5 class a similar degree of shrinkage to the mean (average absolute bias of 0.05) was observed as in the condition with *P* = 0 (comparing Fig. 2d and e), indicating that the bias of the estimates for this group is similar to the bias observed when *P* = 0. For persons from the IRTree class, the estimates of *𝜃*_1_ show more shrinkage towards the mean (average absolute bias of 0.11), which may be due to the lower number of informative responses available per person.

##### Equal class sizes

For *P* = .5, the gPCM-5 shows a larger average absolute bias (0.12) and MSE (0.09) than the mixture model (0.06 and 0.06, respectively), while the average variance was similar (0.05 for both models). As can be seen in Fig. 2c and f, for persons whose true class membership is the IRTree, using the nonmixture gPCM-5 model resulted in *𝜃*_1_ being highly overestimated on the lower end and underestimated at the higher end of the *𝜃*_1_-scale (absolute bias of 0.19). Figure 3c and f show similar patterns for the MSEs.

The estimates of *𝜃*_1_ for persons whose true membership is gPCM-5 were only slightly biased under the gPCM-5 (absolute bias of 0.04), but the direction of the bias is different compared to *P* = 0: For *P* = .5 there is *underestimation* on the lower end and *overestimation* on the higher end of the scale. Thus, instead of the shrinkage towards the mean observed for *P* = 0, the estimates are slightly inflated under the gPCM-5 when *P* = .5 for persons belonging to the gPCM-5 class, indicating that when using the nonmixture model the estimation of *𝜃*_1_ is also not robust for persons for whom a gPCM-5 model would in fact be appropriate.

In contrast, when using the mixture model the bias of the estimates for persons belonging to the gPCM-5 class obtained in this condition is very similar (both in direction and size) to that observed when *P* = 0 (see Fig. 2f and d). Additionally, there is much less discrepancy between the MSEs obtained for persons belonging to the IRTree class and persons belonging to the gPCM-5 class when using the mixture model (Fig. 3f), compared to when the nonmixture gPCM-5 model is used (Fig. 3c).

## Empirical example

The mixture model was applied to data on the ‘Experiences in Close Relationships’ (ECR) questionnaire developed by Brennan, Clark, and Shaver (1998). The questionnaire consists of 36 items belonging to two dimensions (18 items each). The first dimension captures avoidance in close relationships, for example using the item “I don’t feel comfortable opening up to romantic partners”. The second dimension captures anxiety in close relationships, for example using the item “I worry about being abandoned”. The authors derived these items based on a factor analysis using several existing self-report measures of adult romantic relationships. The authors reported that the subtests have Cronbach’s alpha of .94 and .91 for avoidance and anxiety, respectively. Furthermore, in the paper proposing this measurement instrument it was shown that the two dimensions can predict theoretically appropriate target variables (Brennan et al., 1998). All items were five-category Likert items, where the middle category was labeled “neither agree nor disagree”. While this formulation should suggest to the respondent that the middle category belongs to the same scale as the other categories, this does not guarantee that every respondent would use the middle category in this way, and differential use can be investigated using the mixture model. Responses of 1000 persons randomly sampled from a larger sample were used for the analysis.

The mixture model was estimated using the Gibbs Sampler with 10 chains with 10000 iterations each (including 5000 iterations of burn-in). With respect to the number of iterations, we decided to stay on the safe side compared to the simulation study by taking both a longer burn-in and using more iterations for the post-burn-in, because computational time is less of an issue when only one data set needs to be analyzed.

In addition to the mixture model, two non-mixture models were also considered: the gPCM-5 and the IRTree model, both assuming that a single measurement model captures the structure in the data (i.e., assuming IRT MI). Both models were estimated using the same estimation procedure as for the mixture model, but where for all persons class membership was fixed to that of the model that was considered. The relative fit of the three models was compared using the deviance information criterion (DIC), which was used because it adequately takes the complexity of hierarchical models into account (Spiegelhalter, Best, Carlin, & van der Linde, 2002). Model complexity is captured by the number of effective parameters *p*_*D*_, defined as the difference between the deviance averaged across iterations and the deviance computed for the parameter estimates. In hierarchical models *p*_*D*_ is typically smaller than the number of parameters present in the model, because the contribution of a parameter to *p*_*D*_ depends on the ratio of the information about the parameter in the likelihood to its posterior precision (Spiegelhalter, Best, Carlin, & Van der Linde, 1998).

The mixture model performed better than the other two models in terms of the DIC (see Table 3). The mixture model’s complexity (*p*_*D*_) was higher than that of the gPCM-5, but lower than that of the IRTree model, where *𝜃*_0_ is estimated for every person. The mixture model has a lower *p*_*D*_ than the nonmixture IRTree model due to the fact that in the former *𝜃*_0_s do not contribute (or hardly contribute) to *p*_*D*_ for the persons who are classified in the gPCM-5 class with high certainty, because for these persons *𝜃*_*p*0_ is effectively sampled from the prior and is not informed by the data. The fit of the mixture model ($\bar {D}$) was much better than that of the other two models, outweighing (as indicated by the DIC) the increase in complexity in switching from the gPCM-5 model to the mixture model. These results indicate that using the mixture model rather than either one of the two non-mixture models may be preferred.

**Table 3:** Model comparison for the three models fitted to the experience in close relationships data: Expectation of the deviance ($\bar {D}$; measure of model fit), effective number of parameters (*p*_*D*_; measure of model complexity), and deviance information criterion (DIC)

Model	$\bar {D}$	*p*_*D*_	DIC
gPCM-5	86993.17	2010.87	89004.04
IRTree	86441.34	2633.07	89074.41
Mixture	82275.47	2294.56	84570.03

**Fig. 4:**
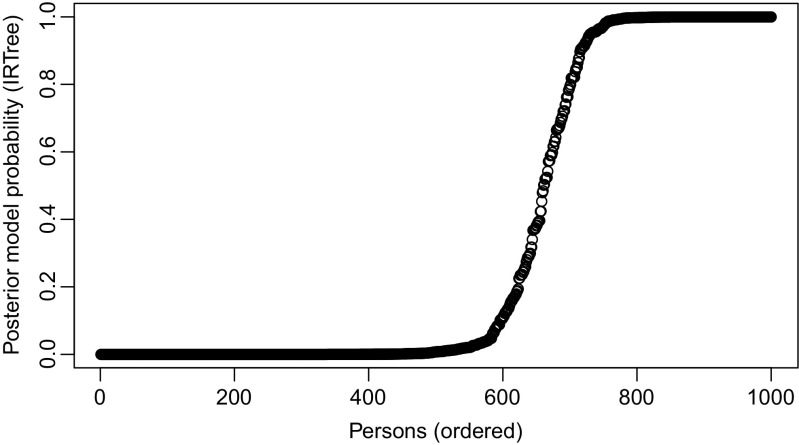
Posterior probabilities of belonging to the IRTree class for the Experience in close relationships data. Each point represents a person, with persons ordered based on their posterior class probability

For the mixture model, based on the estimates of the *Z*_*p*_s, 340 persons were assigned to the IRTree class, and 660 persons were assigned to the gPCM-5 class. Figure 4 shows the estimated posterior probabilities of belonging to the IRTree class with persons ordered based on this probability. Most of the persons were assigned to one of the two classes with high certainty. Among the persons assigned to the IRTree class and to the gPCM-4 class, 79*%* and 88%, respectively, had a posterior probability of belonging to the corresponding class higher than .95.

To investigate whether it is plausible that the improved fit that was obtained when using the mixture model instead of either nonmixture model was due to working with structurally different measurement models in the two classes, we performed a post-hoc analysis in which for each obtained class separately we fitted both the IRTree model and the gPCM-5, and compared both models in terms of fit and DIC. The idea behind this post-hoc analysis was that if a single measurement model would have been appropriate in both of the two classes, the DIC should indicate that measurement model to be preferred in both classes. As can be observed in Table 4, for the group of persons that were assigned to the IRTree class by the mixture model, fitting a gPCM-5 instead of an IRTree model greatly worsens the fit, and the DIC indicates that the IRTree model is much preferred for this class. Vice versa, for the group of persons assigned to the gPCM-5 class fit is worsened when the IRTree model rather than the gPCM-5 is fitted to the data, and the DIC indicates that for this class the gPCM-5 is preferred. These results suggest that using these structurally different measurement models for the two classes is indeed necessary to adequately model the response data.

**Table 4:** Model comparison of the gPCM-5 and IRTree model considered separately for both classes obtained based on the mixture model

	Class *Z* = 0: IRTree			Class *Z* = 1: gPCM		
Model	$\hat {D}$	*p*_*D*_	DIC	$\hat {D}$	*p*_*D*_	DIC
gPCM-5	30361.66	771.84	31133.50	52484.74	1370.02	53854.75
IRTree	29715.09	994.53	30709.63	52671.08	1749.36	54420.44

Consideration of the parameter estimates obtained using the mixture model yielded substantively relevant results that would have been unavailable if only a gPCM-5 would have been used. For the mixture model the median of the estimated *α*_*iS*_s was equal to 0.70, with an interquartile range of [0.55;1.26]. Thus, while the items were not designed to measure persons’ tendencies to avoid giving informative answers (i.e., selecting the middle category when this is interpreted as a nonresponse option), the items do reasonably well in differentiating persons on this tendency to produce noninformative responses (*𝜃*_0_) for persons belonging to the IRTree class, and—as indicated by the interquartile range of the *α*_*iS*_s – the items also differ in the extent to which they capture this tendency. Furthermore, the item location parameter *δ*_*iS*_ showed notable variance across items (standard deviation of 0.76), and ranged from 0.82 to 3.94 (mean of 2.13). This indicates that there was a substantial difference between the items in terms of how likely persons were to provide a noninformative response. While investigating whether certain item properties can be linked to higher or lower values of *δ*_*iS*_ is beyond the scope of this example, such study might be of interest to test constructors who likely want to avoid designing items that evoke noninformative responses.

Interestingly, the correlation between *𝜃*_0_ and the ‘avoidance’ dimension of the ECR is estimated to be .27 (with [.14,.40] as the 95*%* credible interval). This indicates that persons who show a high degree of avoidance in close relationships also display a stronger tendency to avoid giving informative responses, at least on this questionnaire about close relationships. This can be seen as providing some indication that *𝜃*_0_ might capture a substantively relevant dimension that can be related to other relevant person attributes, such as the avoidance tendency that the scale was designed to measure. These findings invite further research into the nature of the tendency to produce noninformative responses as captured by *𝜃*_0_ and its relation to other traits.

## Discussion

### The mixture model for Likert data

This manuscript considered the application of the proposed general mixture IRT framework to address between-person differences in how the middle response category on Likert-scale items is interpreted and used. For this, a mixture of a gPCM and an IRTree model was used, where the IRTree model assumes a person-specific ‘information-avoidance tendency’ to influence the usage of the middle response category. By using a mixture of two structurally different measurement models, the model can accommodate the possibility that persons show qualitative differences in their usage of this category and take this into account for the measurement of the attribute(s) of interest.

It may be noted that our approach to modeling the usage of the middle category in Likert-scale items is distinct from but related to other approaches that consider response styles and nonresponse choice (Raaijmakers et al., 2000; Moors, 2008). That is, like others have suggested before, we relate differential usage of the middle category on Likert-scale items to a person-specific response tendency that is assumed to be continuous, but we predict that the way this tendency displays itself will depend on the interpretation of the response categories: Only if a person considers the middle response category to constitute a viable nonresponse option will its usage depend on that person’s tendency to provide a noninformative response. As a consequence, a person’s response tendency cannot simply be assessed by considering differential use of the middle category, but rather requires us to model a person’s interpretation of that middle category.

The simulation study suggested that if such between-person differences in interpretation and use of the middle category exist, using this mixture model can notably reduce the bias in the person estimates. The biggest gain was obtained for persons belonging to the IRTree class, for whom the gPCM-5 was not the correct model, resulting in severe bias when the mixture was not taken into account. However, the estimates obtained for persons in the gPCM-5 class improved as well, due to improved recovery of the gPCM-5 item parameters as a consequence of not including persons grouped in the IRTree class for the estimation of those parameters. The results of the simulation study indicate that when the test is not too short, the procedure is able to classify most persons with a high degree of certainty.

The application of the proposed mixture model to empirical data suggested that using such a mixture model can improve measurement in practice, as the mixture model outperformed both non-mixture models. Using the mixture model may also provide relevant additional information about persons (estimates of class membership and information-avoidance tendency) as well as items (the extent to which the item evokes noninformative responses) that may be of interest to researchers or test constructors.

### The general mixture IRT framework

In this article we proposed a general mixture IRT framework that allows researchers to use a mixture of structurally different measurement models. The approach was illustrated in the context of a two-class mixture model for Likert data, but it can readily be applied using any set of measurement models for which Bayesian estimation procedures are available and for which the concurrent estimation of these different models is tractable. Usage of these mixture models may lead to improved recovery of the relevant person parameters (i.e., more accurate information about the attributes of interest) as well as improved understanding of the response processes that are involved (e.g., about the differential use of response categories).

While this manuscript has considered a particular application of the general mixture IRT framework, it is to be expected that the framework can be relevant in a variety of other contexts. Whenever different response processes are expected to play a role for different persons, it may be relevant to consider using a mixture of structurally different measurement models. That is, assuming the same measurement model (albeit with different item parameters) to adequately capture qualitatively different response processes may not be realistic, and a mixture of different models may do more justice to the actual underlying processes and improve measurement. For example, in the context of educational measurement researchers often have to deal with the fact that items on educational tests can be solved using different strategies, some of which may only be known to a subgroup. Likewise, students may differ in their willingness to guess on multiple choice items, or may differ in the way they guess (i.e., random guessing versus informed guessing). The framework may also be useful for dealing with the effects of confounding factors such as dyslexia or test anxiety, which may only play a role for part of the sample. Examples such as these are likely to be present in many other fields as well.

It may be noted that for the successful application of the framework the measurement models should result in differential predictions for the expected response patterns, resulting in differences in the likelihood for individual response patterns. That is, for the different measurement models to be separable, they should be empirically nonequivalent. The larger the differences in prediction are, the more easily persons are assigned to the right class and the more can be gained from using a mixture of measurement models instead of a single model. While the simulation results obtained for the specific mixture model that was considered here were encouraging, more research is needed to provide a complete picture of the general conditions under which the procedure performs well.

As a recommendation, we suggested to consider measurement models linked through the inclusion of a shared set of latent variables. While assuming this weak form of MI is not necessary for the mixture model to be estimable, it has strong appeal from the measurement point of view, as it entails that the same attribute is measured in each class. Whether assuming this weak form of MI is reasonable will need to be assessed in the context of the application at hand, which should be tested empirically (e.g., see Messick, 1989, 1995).

It can be noted that even if the model in each class measures the same attribute, that in itself does not guarantee that the latent variables obtained for these models are on the same scale, an issue that holds for mixture IRT models in general (Paek & Cho, 2015). In our application of the procedure we took as a starting point that the latent variable has the same distribution in both classes (i.e., equal mean and variance), and fixed the two scales through the distribution of the latent variables. This may be defensible when there is no reason to assume that class membership is related to the trait that is measured. However, one can consider creating a common scale through the item-side rather than through the person-side of the model if one suspects class differences in the distribution of the shared latent variable(s).^5^ In deciding which way of fixing the scales is preferable, one will have to consider the plausibility of these different possible constraints.

One limitation of the approach as it was presented is that it assumes that there is a person mixture, rather than a person-by-item mixture. This corresponds to assuming that persons can be assigned to a single class for all items, and precludes the possibility of class-switching across items. In the context of the two-class mixture model for Likert data this may make sense, given that the model is supposed to capture different interpretations of the middle category, which one can assume persist across items. However, if one considers for example different measurement models that are supposed to capture different response styles, or the use of different response strategies, then it may make sense to allow for switching of classes across items. While theoretically appealing, this may turn out to be problematic from a practical point of view, because allowing for person-by-item mixtures means that class membership needs to be estimated separately for each item, based on very little information. It would be interesting to explore whether it can generally be feasible to consider such person-by-item mixtures in practice. A possibly more feasible (but also less flexible) alternative would be to consider person-by-subscale mixtures, where class membership is taken to be fixed within a subset of the items but class switching across subscales is allowed.

### Electronic supplementary material

Below is the link to the electronic supplementary material.

(R 43.7 KB)

(C 10.9 KB)

(R 7.03 KB)

(R 3.46 KB)

## Appendix

### Gibbs sampler for the mixture model for Likert data

Before the parameters can be sampled from their full conditional posterior distributions, initial values need to be specified. Random starting values are used for the person class memberships:

11 $$ {Z^{0}_{p}}\sim Bernoulli(.5), $$

where the superscript 0 denotes that it is the initial value. An identity matrix **I**_*D*+ 1_ is chosen for the covariance matrix of person parameters. The continuous person parameters are sampled independently from $\mathcal {N}(0,1)$. The initial values of all item slopes are equal to 1, and the initial values of all item difficulties in the 2PL are equal to 1. The threshold parameters in the gPCM-*m* and gPCM-(*m* − 1) (except *δ*_*i*1*T*_ and *δ*_*i*1*R*_) are spaced with equal distance on the interval between -1.5 and 1.5:

12 $$\begin{array}{@{}rcl@{}} \boldsymbol{\delta}^{0}_{iR}&=&\{0,-1.5,\left( -1.5+\frac{3}{m-1}\right),\dots,\left( -1.5-\frac{3(m-2)}{m-1}\right),1.5\}, \end{array} $$

13 $$\begin{array}{@{}rcl@{}} \boldsymbol{\delta}^{0}_{iT}&=&\{0,-1.5,\left( -1.5+\frac{3}{m-2}\right),\dots,\left( -1.5-\frac{3(m-3)}{m-2}\right),1.5\}. \end{array} $$

At each iteration the algorithm goes through the following steps:

**Step 1**: For each dimension *d* ∈ [1 : *D*], for each person *p* sample *𝜃*_*p**d*_ from its full conditional posterior (i.e., given the current values of all other parameters): 14 $$ p(\theta_{pd}\,|\, \mathbf{X}_{p},\theta_{p0},\boldsymbol{\theta}_{p(d)},\boldsymbol{\alpha}_{R},\boldsymbol{\alpha}_{T},\boldsymbol{\delta}_{R},\boldsymbol{\delta}_{T}, Z_{p},\boldsymbol{\Sigma}), $$ where **X**_*p*_ is the response vector of person *p*, and ***𝜃***_*p*(*d*)_ is the vector of person parameters of person *p* in dimensions except 0 and *d*. The sampling process at Step 1 differs depending on the class membership of person *p*. If *Z*_*p*_ = 1, then the following scheme is used: First, generate a candidate value *𝜃*^∗^ from 15 $$ p(\theta_{pd}\,|\, \theta_{p0},\boldsymbol{\theta}_{p(d)}, \boldsymbol{\Sigma})= \mathcal{N}(\mu^{*}_{d},\sigma_{d}^{*2}), $$ where $\mu ^{*}_{d}$ and $\sigma ^{*2}_{d}$ are the conditional mean and the conditional variance given ***𝜃***_*p*(*d*)_. Second, generate a vector of responses to the items in dimension *d*, denoted by **Y**, according to the gPCM-*m* (see Equation 1) given *𝜃*^∗^ and the current values of ***α***_*R*_ and ***δ***_*R*_. Third, generate a random number $u\sim \mathcal {U}(0,1)$. The candidate value *𝜃*^∗^ is accepted as the new value of *𝜃*_*p**d*_ if 16 $$ u < \exp\left( (\theta^{*}-\theta_{pd}) \left( \sum\limits_{i\in I_{d}} \alpha_{iR} (X_{pi} - Y_{i})\right)\right), $$ where *I*_*d*_ denotes the set of items in dimension *d*. If *Z*_*p*_ = 0: First, generate a candidate value *𝜃*^∗^ from: $ \mathcal {N}(\mu ^{*}_{d},\sigma _{d}^{*2})$, Second, generate a vector of responses to the items in dimension *d*, denoted by **Y**^∗∗^, according to the gPCM-(*m* − 1) (see Eq. 4) given *𝜃*^∗^ and the current values of ***α***_*T*_ and ***δ***_*T*_. Third, generate a random number $u\sim \mathcal {U}(0,1)$. The candidate value *𝜃*^∗^ is accepted as the new value of *𝜃*_*p**d*_ if 17 $$ u \!<\! \exp\left( \!(\theta^{*}\,-\,\theta_{pd})\left( \sum\limits_{i\in I_{d}} (1\,-\,X^{*}_{pi})\alpha_{iT} (X^{**}_{pi} \,-\,Y_{i}^{**})\!\right)\!\right).\!\! $$
**Step 2**: For each person *p* sample *𝜃*_*p*0_ from its full conditional posterior: 18 $$ p(\theta_{p0}\,|\, \mathbf{X}_{p},\boldsymbol{\theta}_{p},\boldsymbol{\alpha}_{S},\boldsymbol{\delta}_{S},Z_{p},\boldsymbol{\Sigma}). $$ For *p* ∈{*p* : *Z*_*p*_ = 1} sample from: $ \theta _{p0}\sim \mathcal {N}\left (\mu ^{*}_{0},\sigma _{0}^{*2}\right ). $ The posterior distribution does not depend on the data for the persons *p* ∈{*p* : *Z*_*p*_ = 1}. For *p* ∈{*p* : *Z*_*p*_ = 0}: First, generate a candidate value from: $\theta ^{*}\sim \mathcal {N}\left (\mu ^{*}_{0},\sigma _{0}^{*2}\right )$. Second, generate a vector of responses to all items, denoted by **Y**^∗^, according to the 2PL (see Eq. 3) given *𝜃*^∗^ and the current values of ***α***_*S*_ and ***δ***_*S*_. Third, generate a random number $u\sim \mathcal {U}(0,1)$. The candidate value *𝜃*^∗^ is accepted as the new value of the parameter if 19 $$ u < \exp\left( (\theta^{*}-\theta_{p0})\left( {\sum}_{i} \alpha_{iS}(X^{*}_{pi} -Y^{*}_{i})\right)\right). $$ where *𝜃*_*p*0_ is the current value of the parameter. Otherwise, the current value is retained.
**Step 3**: For each person *p* sample the person’s class membership *Z*_*p*_ from its full conditional posterior 20 $$ p(Z_{p}\,|\, \mathbf{X}_{p},\theta_{p0},\boldsymbol{\theta}_{p},\boldsymbol{\alpha}_{R},\boldsymbol{\alpha}_{S},\boldsymbol{\alpha}_{T},\boldsymbol{\delta}_{R}, \boldsymbol{\delta}_{S},\boldsymbol{\delta}_{T},\pi), $$ which is a Bernoulli distribution with the probability: 21 $$ \Pr(Z_{p}= 1)=\frac{1}{1+O_{p}}, $$ where 22 $$ O_{p}=\frac{(1-\pi)}{\pi}\frac{ {\prod}_{i} h_{1}\left( X^{*}_{pi}\,|\, \theta_{p0},\alpha_{iS},\delta_{iS}\right) \left( h_{2}(X^{**}_{pi}\,|\,\theta_{pd_{i}},\alpha_{iT},\boldsymbol{\delta}_{iT})\right)^{1-X^{*}_{pi}} }{{\prod}_{i} g(X_{pi}\,|\, \theta_{pd_{i}},\alpha_{iR},\boldsymbol{\delta}_{iR}) } $$ are the posterior odds of belonging to the IRTree class, which are the product of the prior odds and the ratio of likelihoods of **X**_*p*_ under the two models.
**Step 4**: Sample *π* from its full conditional posterior *p*(*π*| **Z**) which depends only on the current class memberships of the persons. This conditional posterior is a beta distribution: 23 $\mathcal{B}\left( 1+\sum\limits_{p}Z_{p},1+N-\sum\limits_{p} Z_{p}\right).$
**Steps 5–7**: For each item *i* sample its slope parameters *α*_*i**R*_, *α*_*i**S*_ and *α*_*i**T*_ from their full conditional posterior distributions: 24 $$\begin{array}{@{}rcl@{}} p(\alpha_{iR}\,|\, \mathbf{X}_{i},\boldsymbol{\theta}_{d_{i}},\boldsymbol{\delta}_{iR}, \mathbf{Z})&\propto& \,p(\alpha_{iR})\prod\limits_{p} \left( g(X_{pi}\,|\, \theta_{pd_{i}}, \alpha_{iR}, \boldsymbol{\delta}_{iR})\right)^{Z_{p}}, \end{array} $$ 25 $$\begin{array}{@{}rcl@{}} p(\alpha_{iS}\,|\, \mathbf{X}_{i},\boldsymbol{\theta}_{0},\delta_{iS}, \mathbf{Z})&\propto& \,p(\alpha_{iS})\prod\limits_{p} \left( h_{1}\left( X^{*}_{pi}\,|\, \theta_{p0},\alpha_{iS},\delta_{iS}\right)\right)^{1-Z_{p}}, \end{array} $$ 26 $$\begin{array}{@{}rcl@{}} p(\alpha_{iT}\,|\, \mathbf{X}_{i},\boldsymbol{\theta}_{d_{i}},\boldsymbol{\delta}_{iT}, \mathbf{Z})&\propto & \,p(\alpha_{iT})\prod\limits_{p} \left( h_{2}\left( X^{**}_{pi}\,|\, \theta_{pd_{i}},\alpha_{iT},\boldsymbol{\delta}_{iS}\right)\right)^{(1-X^{*}_{pi})(1-Z_{p})}, \end{array} $$ where **X**_*i*_ is a vector of responses of all persons to item *i*, $\boldsymbol {\theta }_{d_{i}}$ is a vector of person parameters of all persons on dimension *d*_*i*_, ***𝜃***_0_ is the vector of person parameters of all persons in the first node of the IRTree. Sampling from each of these posteriors is done using a Random walk Metropolis algorithm with a log-normal proposal centered around the log of the current value (Metropolis, Rosenbluth, Rosenbluth, Teller, & Teller, 1953; Hastings, 1970).
**Steps 8–10**: For each item *i* sample its threshold parameters ***δ***_*i**R*_, ***δ***_*i**T*_ and the difficulty *δ*_*i**S*_ from their full conditional posterior distributions. To make the sampling procedure more stable, instead of sampling the threshold parameters we sample the intercept parameters: 27 $$\begin{array}{@{}rcl@{}} \beta_{ikR}&=&-\alpha_{iT}\delta_{ikT}, \forall k\in[2:m], \end{array} $$ 28 $$\begin{array}{@{}rcl@{}} \beta_{iS}&=&-\alpha_{iS}\delta_{iS}, \end{array} $$ 29 $$\begin{array}{@{}rcl@{}} \beta_{ikR}&=&-\alpha_{iR}\delta_{ikR},\forall k\in [2:(m-1)]. \end{array} $$ from their full conditional posteriors 30 $$\begin{array}{@{}rcl@{}} p(\beta_{ikR}\,|\, \mathbf{X}_{i},\boldsymbol{\theta}_{d_{i}}, \alpha_{iR},\boldsymbol{\delta}_{i(k)R}, \mathbf{Z}), &\forall& k\in[2:m], \end{array} $$ 31 $$\begin{array}{@{}rcl@{}} p(\beta_{iS}\,|\, \mathbf{X}_{i},\boldsymbol{\theta}_{0},\alpha_{iS}, \mathbf{Z}),&& \end{array} $$ 32 $$\begin{array}{@{}rcl@{}} p(\beta_{ikT}\,|\, \mathbf{X}_{i},\boldsymbol{\theta}_{d_{i}}, \alpha_{iT},\boldsymbol{\delta}_{i(k)T}, \mathbf{Z}),&\forall& \!k\!\in[2:(m\,-\,1)], \end{array} $$ where ***δ***_*i*(*k*)*R*_ and ***δ***_*i*(*k*)*T*_ are vectors of all the item threshold parameters of item *i* except the *k*-th threshold in the gPCM-*m* and the gPCM-(*m* − 1), respectively. Sampling from each of the posteriors is done using a Random walk Metropolis algorithm with a normal proposal centered around the current value. It may be noted that due to the transformation the priors of the intercept parameters are not independent of the slope parameters: 33 $$\begin{array}{@{}rcl@{}} p(\beta_{ikT}\,|\, \alpha_{iT})&=&\mathcal{N}(\beta_{ikT}; 0,10\alpha_{iT}^{2}) \end{array} $$ 34 $$\begin{array}{@{}rcl@{}} p(\beta_{iS}\,|\, \alpha_{iS})&=&\mathcal{N}(\beta_{iS}; 0,10\alpha_{iS}^{2}) \end{array} $$ 35 $$\begin{array}{@{}rcl@{}} p(\beta_{ikR}\,|\, \alpha_{iR})&=&\mathcal{N}(\beta_{ikR}; 0,10\alpha_{iR}^{2}) \end{array} $$ The sampled parameters are then transformed back to the original parameters.
**Step 12**: Sample the covariance matrix of the person parameters from its full conditional posterior *p*(**Σ** | ***𝜃***), which given the inverse-Wishart prior is known to be an inverse-Wishart distribution (Hoff, 2009): 36 $\boldsymbol{\Sigma}\sim \mathcal{I}\mathcal{W}\left( \mathbf{I}_{D + 1}+\sum\limits_{p} (\theta_{p0},\boldsymbol{\theta}_{p})(\theta_{p0},\boldsymbol{\theta}_{p})^{T},D + 3+N \right).$
**Step 13**: Because in IRT models only the product of the slope parameter and the person parameter is identified, at each iteration we re-scale the model parameters to equate the variances of the person parameters to 1: 37 $$ \begin{array}{rcll} \theta_{pd}&\rightarrow&\frac{\theta_{pd}}{\sqrt{{\Sigma}_{dd}}},&\forall p\in [1:N],\forall d\in[0:D]\\ \alpha_{iR}&\rightarrow&\alpha_{iR}\sqrt{{\Sigma}_{d_{i}d_{i}}},& \forall i\in [1:n]\\ \alpha_{iS}&\rightarrow&\alpha_{iS}\sqrt{{\Sigma}_{00}},& \forall i\in [1:n]\\ \alpha_{iT}&\rightarrow&\alpha_{iT}\sqrt{{\Sigma}_{d_{i}d_{i}}},& \forall i\in [1:n]\\ {\Sigma}_{de}&\rightarrow&\frac{{\Sigma}_{de}}{\sqrt{{\Sigma}_{dd}}\sqrt{{\Sigma}_{ee}}},& \forall d,e\in[0:D] \end{array} $$ At the initialization the starting values of the person and item parameters might be far from where the posterior mass is concentrated. To make sure that at the beginning of the algorithm one of the classes does not get empty due to the item and the person parameters in that class having suboptimal values after the initialization, in the first 200 iterations Step 3 of the algorithm is omitted. That is, in the first 200 iterations we sample not from the conditionals of the joint posterior in Eq. 8 but from the conditionals of 38 $p(\boldsymbol{\theta}_{0},\boldsymbol{\theta},\boldsymbol{\alpha}_{T},\boldsymbol{\delta}_{T},\boldsymbol{\alpha}_{S},\boldsymbol{\delta}_{S}, \boldsymbol{\alpha}_{R},\boldsymbol{\delta}_{R},\boldsymbol{\Sigma},\pi\,|\, \mathbf{X},\mathbf{Z}^{0}),$ meaning that for the first 200 iterations **Z** does not get updated. After the first 200 iterations the sampled parameters have gotten closer to where the posterior density is concentrated, and the full algorithm starts including sampling of the person class memberships.

## Footnotes

1 It may be noted that the midpoint response style is in some modeling approaches (e.g., see Tutz & Berger, 2016) seen as being the opposite of an extreme response style (i.e., a tendency to use mostly extreme categories), which both are captured using a single dimension.

2 The subscript *R* refers to the “**R** egular” model, because the gPCM-*m* is a simpler and more common model for the Likert data than the IRTree model.

3 It may be noted that because our mixture model considers two measurement models that have a different parametric form, label switching both across chains and within chains of the sampler by definition cannot occur (Jasra, Holmes, & Stephens, 2005). Thus, while label switching can be problematic for approaches dealing with structurally identical models (e.g., standard IRT mixture models), this is not an issue for the current approach.

4 The nonmixture gPCM-5 was estimated using a Gibbs Sampler similar to the one used for estimating the mixture model, but which only considers a single class (i.e., with every person permanently assigned to the gPCM-5 class).

5 In the case of the mixture model for Likert data, one could for example fix the scales through the item-side of the models by constraining the threshold parameter $\bar {\delta _{i}}$ of the gPCM-5 and the gPCM-4 to have the same mean and variance, and freely estimating the distribution of ***𝜃***_*p*_ for one of the classes.
